# Vasohibin-1 rescues erectile function through up-regulation of angiogenic factors in the diabetic mice

**DOI:** 10.1038/s41598-020-80925-7

**Published:** 2021-01-13

**Authors:** Kang-Moon Song, Woo Jean Kim, Min-Ji Choi, Ki-Dong Kwon, Anita Limanjaya, Kalyan Ghatak, Jiyeon Ock, Guo Nan Yin, Yasufumi Sato, Soon-Sun Hong, Ji-Kan Ryu, Jun-Kyu Suh

**Affiliations:** 1 National Research Center for Sexual Medicine and Department of Urology, Inha University School of Medicine, 27, Inhang-Ro, Jung-Gu, Incheon, 22332 Republic of Korea; 2 Department of Anatomy, Kosin University College of Medicine, Busan, 49267 Republic of Korea; 3 Department of Vascular Biology, Institute of Development, Aging and Cancer, Tohoku University, Sendai, 980-8575 Japan; 4 Department of Drug Development, Inha University School of Medicine, Incheon, 22332 Republic of Korea

**Keywords:** Molecular medicine, Urology

## Abstract

Neovascularization of the erectile tissue emerges as a beneficial curative approach to treat erectile dysfunction (ED). Here we for the first time report the unexpected role of vasohibin-1 (VASH1), mainly known as an anti-angiogenic factor, in restoring erectile function in diabetic mice. A diabetic patient has lower cavernous VASH1 expression than in the potent man. VASH1 was mainly expressed in endothelial cells. There were significant decreases in cavernous endothelial cell and pericyte contents in VASH1 knockout mice compared with those in wild-type mice, which resulted in impairments in erectile function. Intracavernous injection of VASH1 protein successfully restored erectile function in the diabetic mice (~ 90% of control values). VASH1 protein reinstated endothelial cells, pericytes, and endothelial cell–cell junction proteins and induced phosphorylation of eNOS (Ser1177) in the diabetic mice. The induction of angiogenic factors, such as angiopoietin-1 and vascular endothelial growth factor, is responsible for cavernous angiogenesis and the restoration of erectile function mediated by VASH1. Altogether, these findings suggest that VASH1 is proangiogenic in diabetic penis and is a new potential target for diabetic ED.

## Introduction

Erectile dysfunction (ED) is a mainly vascular disease and erectile tissue has a distinct vascular system^1^. It was reported that more than 50% of the diabetic patients eventually have ED^2^. Moreover, ED is the first sign in some population of the diabetic patients^3^. The main pathophysiologic causes of diabetic ED are structural and functional derangements of cellular components of erectile tissue, such as pericytes, smooth muscle cells, and endothelial cells^4, 5^. The degree of cavernous microangiopathy and a decrease in bioavailable endogenous nitric oxide (NO) from diabetes is related to low responsiveness to oral phosphodiesterase-5 inhibitors^6, 7^.

Vasculogenic ED demands an effective therapeutic approach to rebuild the structural and functional integrity of cavernous microvasculature. Previous animal studies revealed that local delivery of angiogenic factors, such as basic fibroblast growth factor (bFGF), angiopoietin-1 (Ang1), vascular endothelial growth factor (VEGF), and angiopoietin-4 (Ang4), rescued the erectile function in diabetic condition^8–12^. These findings provide us a proof-of-concept of therapeutic angiogenesis as a potential therapeutic strategy for diabetic ED.

Vasohibin-1 (VASH1) is originally discovered as an endothelium-derived inhibitor of angiogenesis. In the animal model of diabetic nephropathy and ischemic retinopathy, the upregulation of VASH1 blocked aberrant angiogenic activity induced by VEGF^13, 14^. Meanwhile, VASH1 expression in tumor endothelial cells is positively correlated with microvascular density in a variety of cancers, such as cervical cancer^15^, colorectal cancer^16^, renal cell carcinoma^17^, and prostate cancer^18^, which suggests that VASH1 enhances angiogenesis in the tumor environment.

Vascular complications in the diabetic nephropathy model were more severe in VASH1 heterozygous knockout (VASH1^+/−^) mice than in wild-type mice^19^. Moreover, it was also reported in the cisplatin-induced acute kidney injury model that the peri-tubular capillary loss and the increase in serum creatinine concentrations were more prominent in VASH1^+/−^ mice than in wild-type mice^20^. These findings suggest that endogenous VASH1 is required for the protection of renal blood vessels in pathologic conditions.

In this study, we studied function of VASH1 on cavernous vascular integrity and erectile function by using VASH1 homozygous-knockout (VASH1^−/−^) mice or the streptozotocin (STZ)-induced diabetic mice.

## Results

### Physiologic and metabolic parameters

Bodyweight, mean systolic blood pressure (MSBP), and blood glucose level of each experimental group were summarized in Supplementary Table 1–3.

### VASH1 is mainly expressed in cavernous endothelial cells

**Figure 1:**
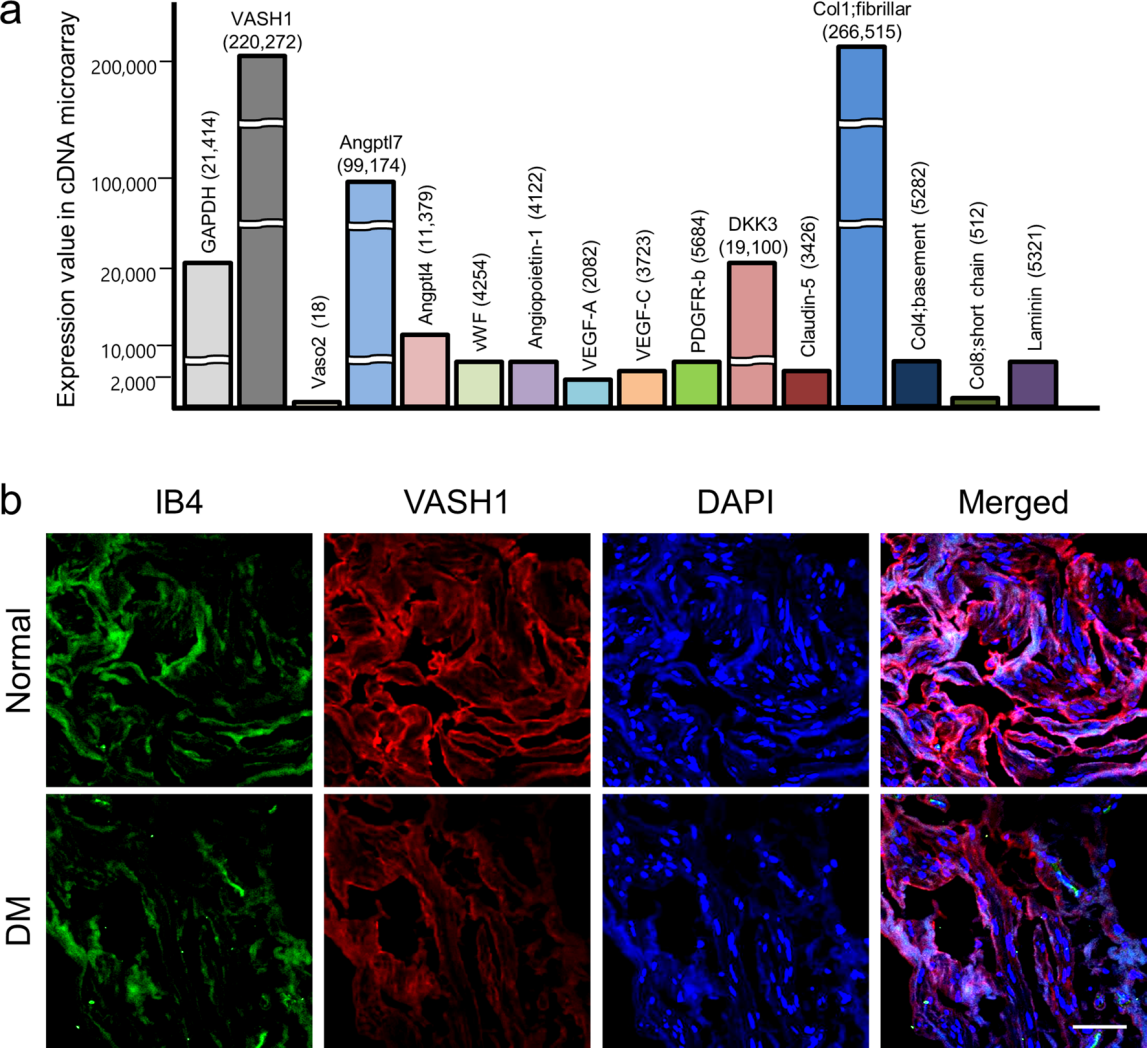
Decreased VASH1 expression under diabetic condition. (**a**) cDNA microarray from mouse corpus cavernosum tissue. The genes that are highly expressed in the erectile tissue has been summarized. (**b**) VASH1 (red) and isolectin IB4 (an endothelial cell marker, green) staining in cavernous tissue from a patient with diabetic erectile dysfunction (IIEF-5 score = 10) or a man with normal potency (IIEF-5 score = 23). Note a significant expression of VASH1 in the endothelial layer. Scale bar = 50 µm. *DM *diabetes mellitus, *IIEF-5 *5-item version of the International Index of Erectile Function.

The cDNA microarray of mouse corpus cavernosum tissue was performed as a screening approach for the prediction of new therapeutic target(s) or candidate(s) to treat diabetic ED. We found that several growth factors, such as VASH1, angiopoietin-like protein 4, dickkopf3, and angiopoietin-like protein 7, are relatively abundantly expressed in the erectile tissue. Of those growth factors, we focused on VASH1 that shows exceedingly high expression in the corpus cavernosum (Fig. 1a).

Next, the differential expression of VASH1 in human erectile tissue was determined. Immunohistochemical staining revealed a reduction of VASH1 expression in the corpus cavernosum of the diabetic patient compared to the control group. Co-labeling of the cavernous tissue with antibodies to IB4 (an endothelial cell marker) and VASH1 demonstrated that VASH1 was co-localized mostly with cavernous endothelial cells (Fig. 1b).

### Downregulation of VASH1 decreases cavernous endothelial cell and pericyte contents, and deteriorates penile erection

**Figure 2:**
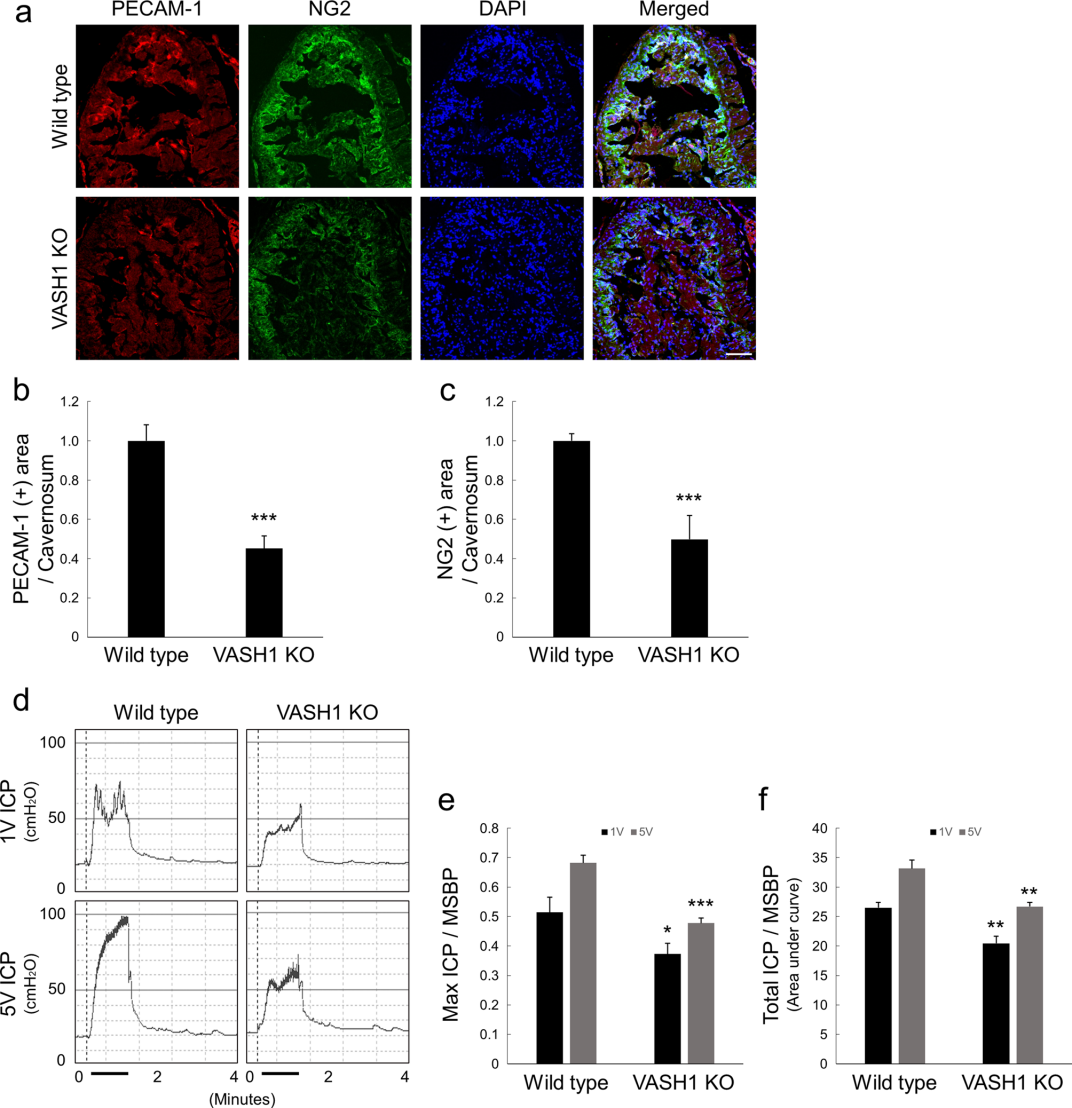
The decrease in cavernous endothelial cell-pericyte contents and impairment of erectile function in VASH1-knockout mice. (**a**) PECAM-1 (red) and NG2 (green) staining in corpus cavernosum of wild-type and VASH1^-/-^ mice. Scale bar = 100 µm. (**b**,**c**) Quantification of cavernous endothelial cell and pericyte content by Image J. Each bar depicts the mean (± SE) values from N = 6 animals per group. ***P < 0.001 vs. wild-type mice. (**d**) Representative intracavernous (ICP) responses for the wild-type and VASH1^−/−^ mice. The stimulus interval is indicated by a solid bar. (**e**,**f**) Ratios of mean maximal ICP and total ICP (area under the curve) to mean systolic blood pressure (MSBP) calculated for each group. Each bar depicts the mean (± SE) values from N = 6 animals per group. *P < 0.05, **P < 0.01, ***P < 0.001 vs. wild-type mice.

Immunohistochemical staining of cavernous tissue with antibody to an endothelial cell maker PECAM-1 or a pericyte marker neuron-glial antigen 2 (NG2) revealed that the cavernous endothelial cell and pericyte content was depressed significantly in VASH1-knockout mice compared with wild-type (WT) mice (Fig. 2a–c). These results imply that VASH1 has a potential protective role in penile vasculature.

Therefore, we analyzed the role of target deletion of VASH1 gene on penile erection. In order to analyze the physiological significance of the downregulation of VASH1, erectile function assessed by in vivo cavernous nerve stimulation. In line with the findings of immunohistochemistry, demonstrating a decline in cavernous endothelial cell and pericyte content in the VASH1-knockout mice, the proportion of maximal intracavernous pressure (ICP) to MSBP, or total ICP to MSBP was significantly lower in VASH1-knockout mice than in WT group (Fig. 2d–f).

### VASH1 protein transfer restores erectile function in the diabetic mice

**Figure 3:**
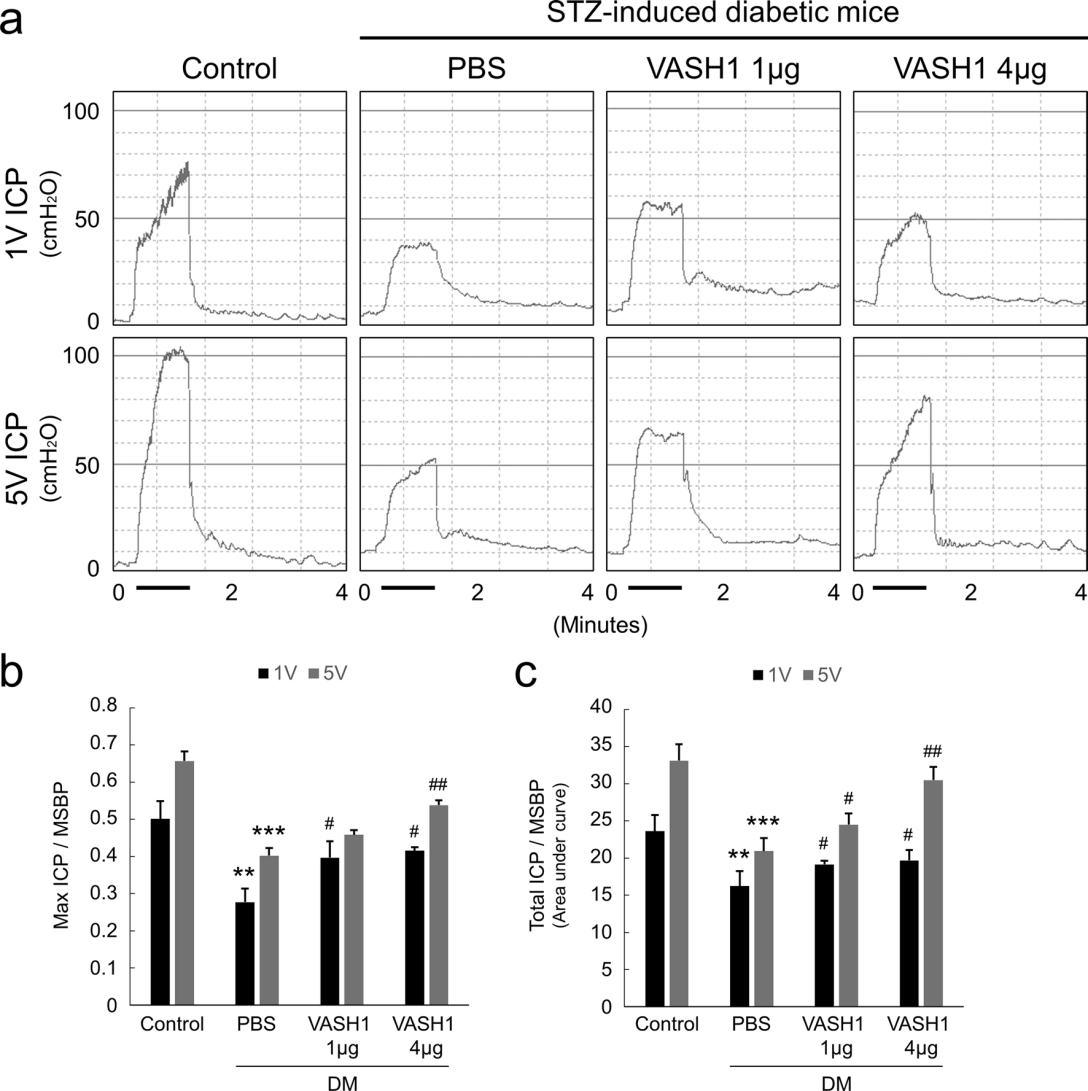
VASH1 protein transfer restores erectile function in diabetic mice. (**a**) Representative intracavernous (ICP) responses for the age-matched control and streptozotocin-induced diabetic mice stimulated at 2 weeks after repeated intracavernous injections of PBS (days − 3 and 0) or VASH1 protein (days − 3 and 0; 1 μg/20 μL or 4 μg/20 μL). The stimulus interval is indicated by a solid bar. (**b**,**c**) Ratios of mean maximal ICP and total ICP (area under the curve) to mean systolic blood pressure (MSBP) calculated for each group. Each bar depicts the mean (± SE) values from N = 6 animals per group. **P < 0.01 and ***P < 0.001 vs. control group. ^#^*P* < 0.05 and ^##^*P* < 0.01 vs. PBS-treated DM group. *DM *diabetes mellitus.

We further defined the impact of VASH1 protein treatment in diabetic mice. The ratios of maximal and total ICP to MSBP in PBS-treated diabetic mice were profoundly decreased compared with that in the control group. The repeated intracavernous injections of VASH1 (4 μg/20 μL) protein (days − 3 and 0) significantly restored erectile function in the diabetic mice. However, VASH1 at a dosage of 1 μg/20 μL didn’t show significant improvements in erectile function (Fig. 3).

### VASH1 protein transfer restores cavernous endothelial cell and pericyte contents in the diabetic mice

**Figure 4:**
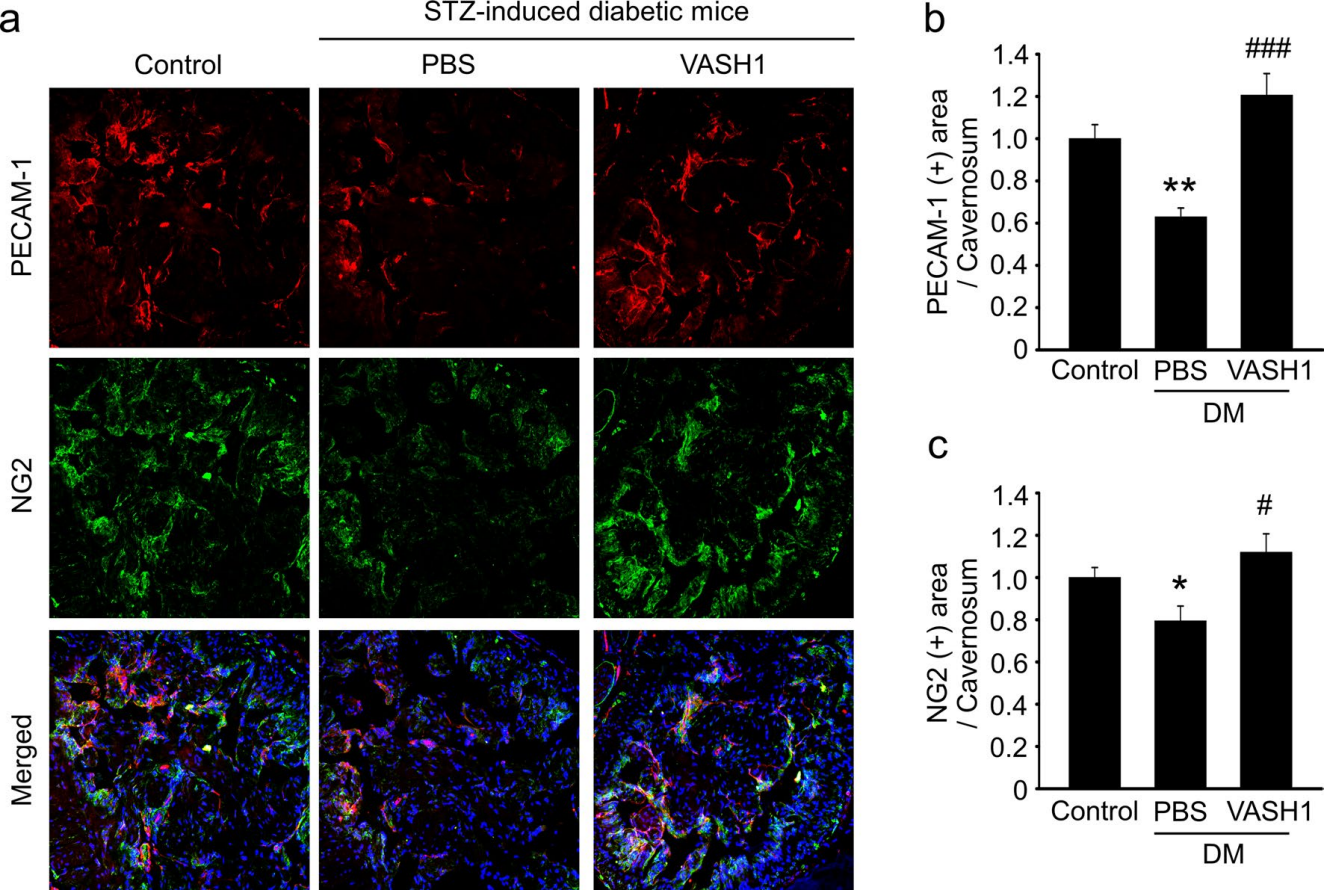
VASH1 protein transfer restores cavernous endothelial cell and pericyte content in diabetic mice. (**a**) PECAM-1 (red) and NG2 (green) staining in cavernous tissue from age-matched control and streptozotocin-induced diabetic mice 2 weeks after receiving repeated intracavernous injections of PBS (days − 3 and 0) or VASH1 protein (days − 3 and 0; 4 μg/20 μL). Scale bar = 100 µm. (**b**,**c**) Quantification of cavernous endothelial cell and pericyte content by Image J. Each bar depicts the mean (± SE) values from N = 6 animals per group. *P < 0.05, **P < 0.01 vs. control group. ^#^*P* < 0.05, ^###^*P* < 0.001 vs. PBS-treated DM group. *DM *diabetes mellitus.

Immunofluorescent staining of cavernous tissue with antibodies to PECAM-1 and NG2 was performed in control or diabetic mice 2 weeks after treatment. We observed lower endothelial cell and pericyte contents in the PBS-treated diabetic group than in the control group. Intracavernous administration of VASH1 protein (days − 3 and 0; 4 μg/20 μL) completely restored cavernous endothelial cell and pericyte contents in the diabetic mice (Fig. 4).

### VASH1 protein transfer restores cavernous endothelial cell to cell junction proteins in the diabetic mice

**Figure 5:**
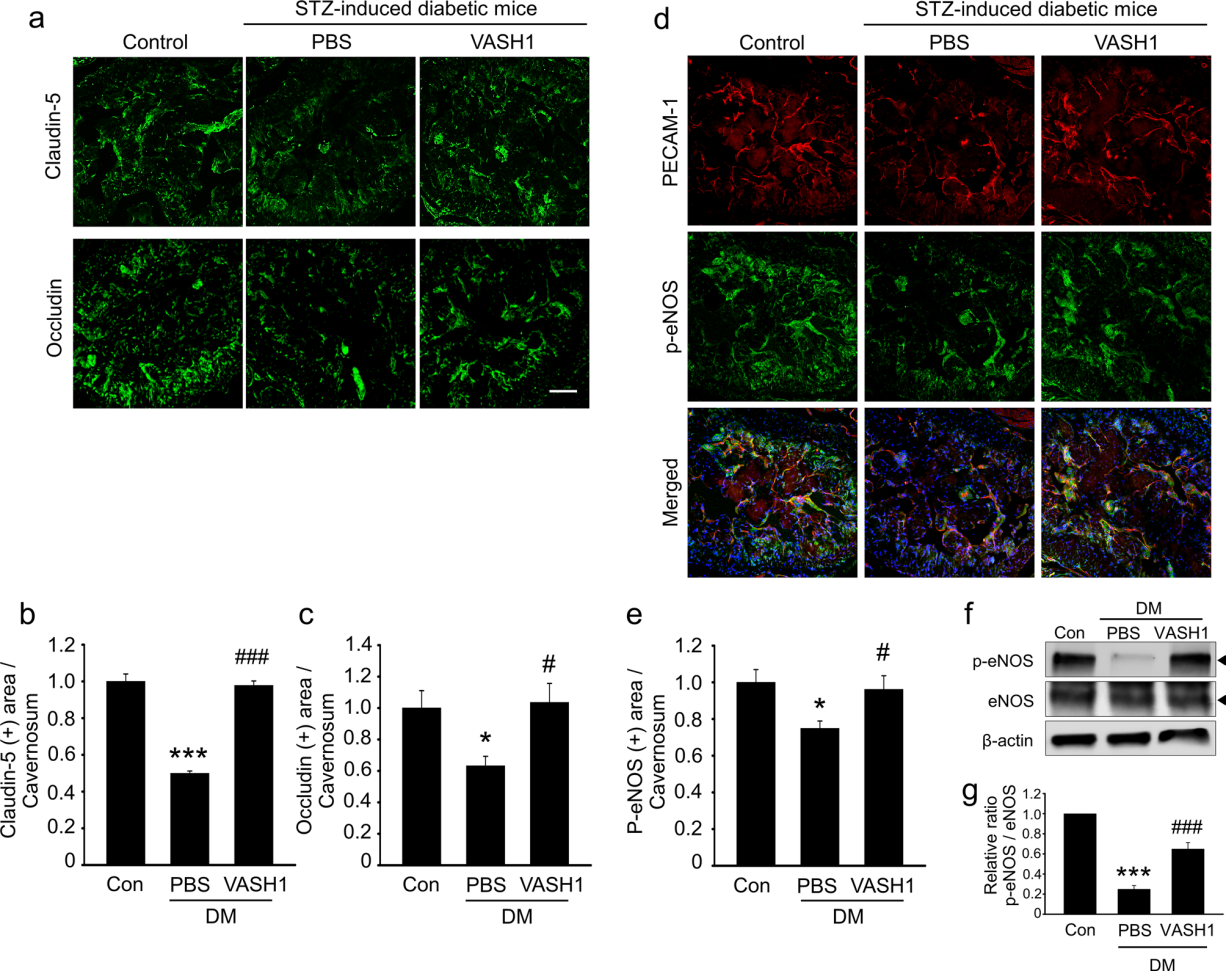
VASH1 protein transfer restores cavernous endothelial cell–cell junction proteins and induces eNOS phosphorylation in diabetic mice. (**a**) Occludin (green) and claudin-5 (green) staining in cavernous tissue from age-matched control and streptozotocin-induced diabetic mice 2 weeks after receiving repeated intracavernous injections of PBS (days − 3 and 0) or VASH1 protein (days − 3 and 0; 4 μg/20 μL). Scale bar = 100 µm. (**b**,**c**) Occludin and claudin-5-immunopositive areas were quantified by Image J. Each bar depicts the mean (± SE) values from N = 6 animals per group. *P < 0.05, ***P < 0.001 vs. control group. ^#^*P* < 0.05, ^###^*P* < 0.001 vs. PBS-treated DM group. (**d**) Phospho-eNOS (p-eNOS, green) and PECAM-1 (red) staining in cavernous tissue from each group of mice. **(e)** P-eNOS-immunopositive areas were quantified by Image J. Each bar depicts the mean (± SE) values from N = 6 animals per group. *P < 0.05 vs. control group. ^#^*P* < 0.05 vs. PBS-treated DM group. (**f**) Representative Western blots for phospho-eNOS and eNOS in each group of mice. (**g**) Relative ratio of phospho-eNOS compared with that of eNOS. Each bar depicts the mean (± SE) values from N = 4 animals per group. ***P < 0.001 vs. control group. ^**###**^*P* < 0.001 vs. PBS-treated DM group. *DM *diabetes mellitus. Full length blot as well as the related images available in the supplementary data.

Endothelial cells adhere to the adjacent ones by endothelial cell to cell junctional proteins, which is important to regulate vascular permeability^21^. Derangements in endothelial cell junctions are known to be a distinctive pathophysiologic mechanism involved in diabetic ED^22^. The expression of endothelial cell to cell junction proteins (occludin and claudin-5) in the cavernous tissue was decreased in the diabetic group treated with PBS compared with that in the control mice. VASH1 protein recovered cavernous endothelial cell to cell junction proteins in diabetic mice, which are equivalent to the levels of the age-matched controls (Fig. 5a–c).

### VASH1 protein transfer induces phosphorylation of endothelial nitric oxide synthase (eNOS) in the diabetic mice

The phosphorylation of eNOS (Ser1177) is a crucial step to produce NO from endothelial cells^23^. eNOS phosphorylation was significantly impaired in the diabetic mice and VASH1 protein successfully induced phosphorylation of eNOS in the corpus cavernosum of diabetic mice (Fig. 5d–g).

### VASH1 protein transfer increases the expression of angiogenic factors in the diabetic mice

**Figure 6:**
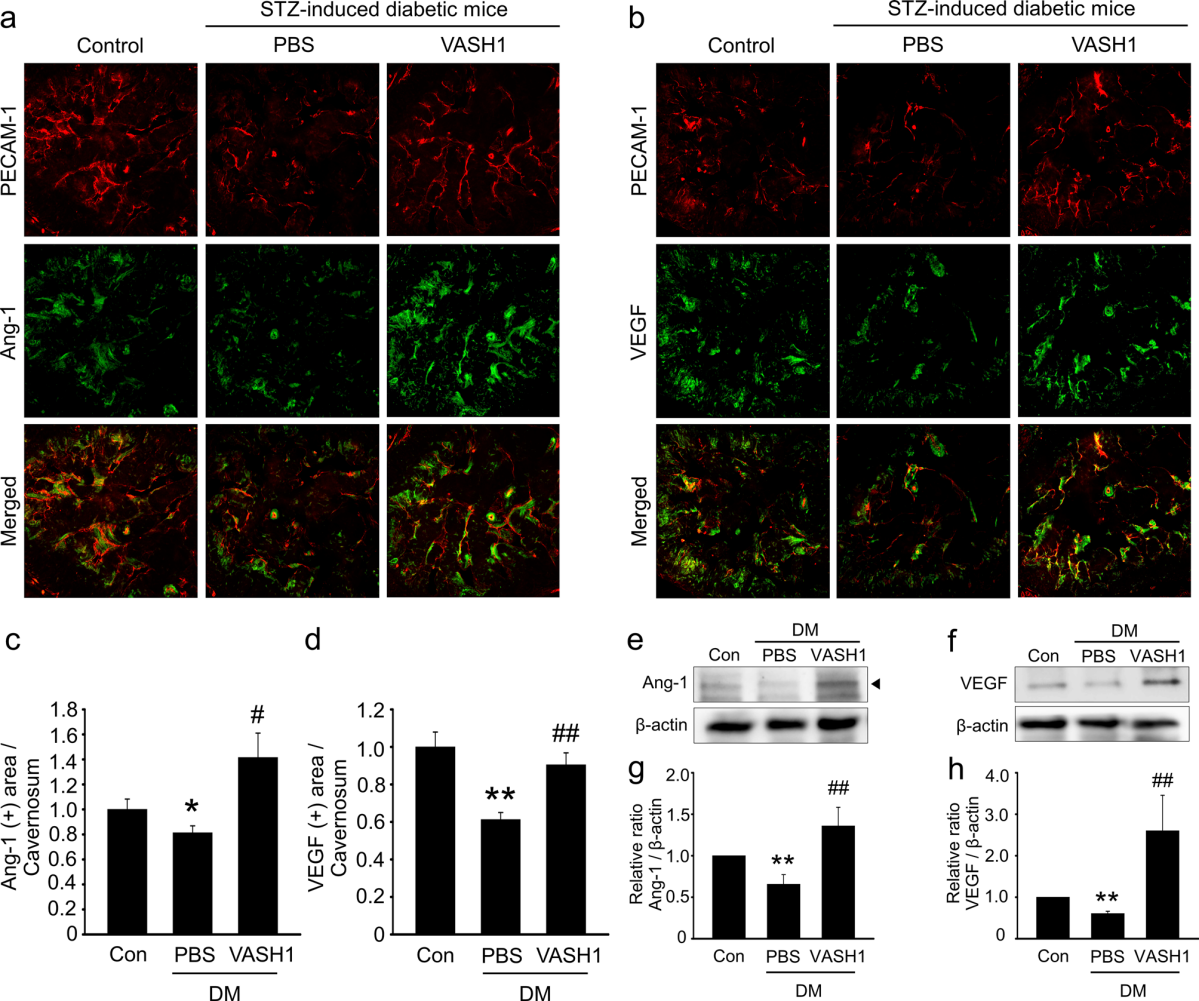
VASH1 protein transfer increases the cavernous expression of angiogenic factors in diabetic mice. (**a**,**b**) Angiopoietin-1 (Ang1, green) and PECAM1 (red), or vascular endothelial growth factor (VEGF, green) and PECAM-1 (red) staining in cavernous tissue from age-matched control and streptozotocin-induced diabetic mice 2 weeks after receiving repeated intracavernous injections of PBS (days − 3 and 0) or VASH1 protein (days − 3 and 0; 4 μg/20 μL). Scale bar = 100 µm. (**c**,**d**) Ang1 and VEGF-immunopositive areas were quantified by Image J. Each bar depicts the mean (± SE) values from N = 6 animals per group. *P < 0.05, **P < 0.01 vs. control group. ^#^*P* < 0.05, ^##^*P* < 0.01 vs. PBS-treated DM group. (**e**,**f**) Representative Western blots for Ang1 and VEGF in each group of mice. (**g**,**h**) The relative ratio of Ang1 and VEGF compared with that of β-actin. Each bar depicts the mean (± SE) values from N = 4 animals per group. **P < 0.01 vs. control group. **#**#*P* < 0.01 vs. PBS-treated DM group. *DM *diabetes mellitus. Full length blot as well as the related images available in the supplementary data.

We performed immunohistochemical staining and Western blot to examine whether the production of angiogenic factors was enhanced by VASH1 protein. The expression of Ang1 and VEGF was significantly lower in the corpus cavernosum of diabetic mice treated with PBS than in the control group. VASH1 protein increased the expression of Ang1 and VEGF in the diabetic mice (Fig. 6).

### Inhibition of Ang1 and VEGF abolishes VASH1 protein-mediated angiogenesis and restoration of erectile function in the diabetic mice

**Figure 7:**
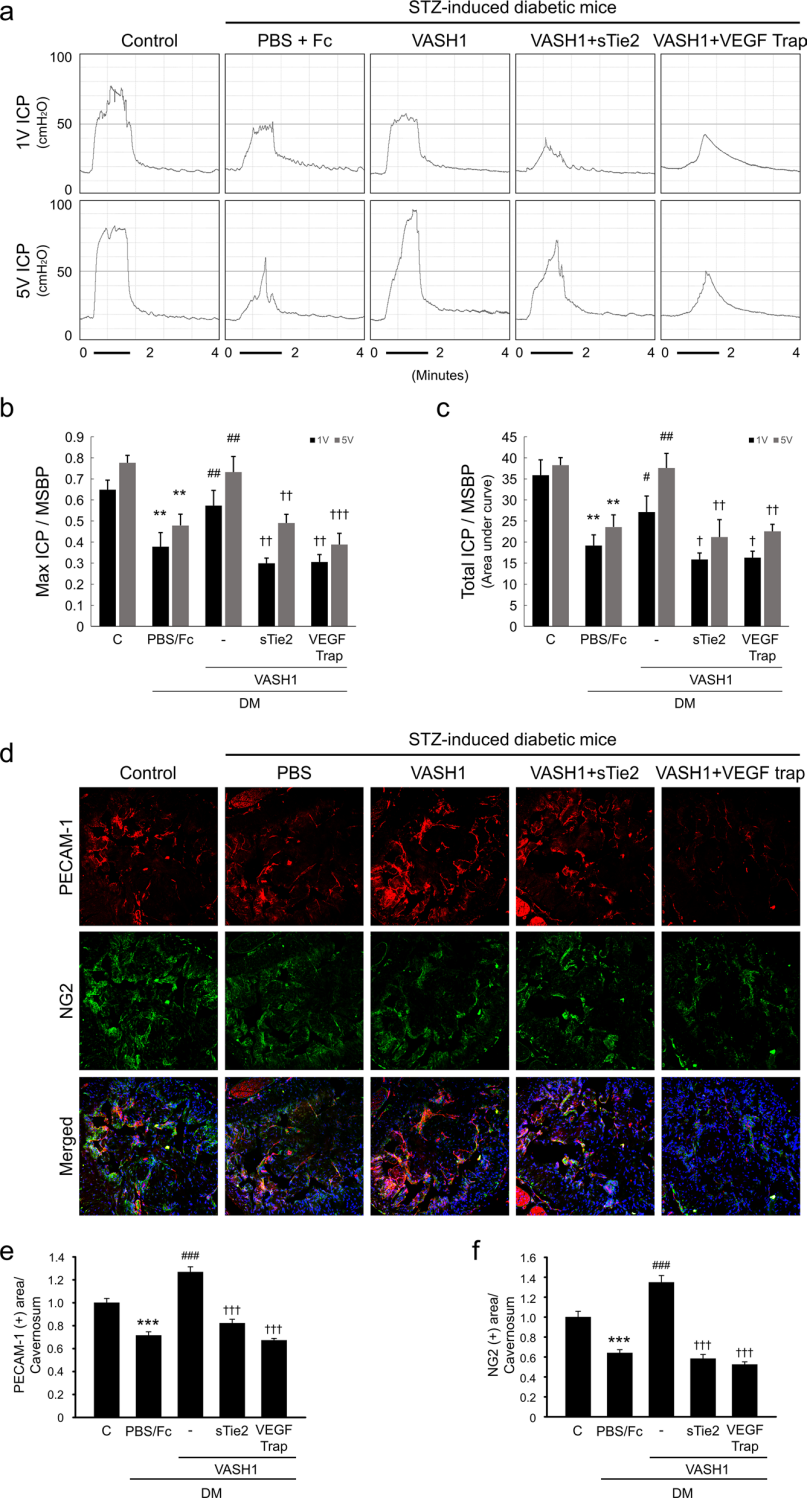
Inhibition of Ang1 and VEGF diminishes VASH1 protein-mediated angiogenesis and recovery of erectile function in the diabetic mice. (**a**) Representative intracavernous (ICP) responses for the age-matched control and streptozotocin-induced diabetic mice stimulated at 2 weeks after repeated intracavernous injections of PBS + dimeric Fc (day − 3 and 0), VASH1 protein (day − 3 and 0; 4 μg/20 μL), VASH1 protein + soluble Tie2-Fc(sTie2-Fc; 4 μg/20 μL), or VASH1 protein + VEGF trap (4 mg/kg in 20 µL PBS). The stimulus interval is indicated by a solid bar. (**b**,**c**) Ratios of mean maximal ICP and total ICP (area under the curve) to mean systolic blood pressure (MSBP) were calculated for each group. Each bar depicts the mean (± SE) values from N = 6 animals per group. **P < 0.01 vs. control group. ^#^*P* < 0.05 and ^##^*P* < 0.01 vs. PBS- + dimeric Fc-treated group. ^†^*P* < 0.05, ^††^*P* < 0.01, and ^†††^*P* < 0.001 vs. VASH1-treated DM group. (**d**) PECAM-1 (red) and NG2 (green) staining in cavernous tissue from each group of animals. Scale bar = 100 µm. (**e**,**f**) Quantification of cavernous endothelial cell and pericyte content by Image J. Each bar depicts the mean (± SE) values from N = 6 animals per group. ***P < 0.001 vs. control group. ^###^*P* < 0.001 vs. PBS- + dimeric Fc-treated group. ^†††^*P* < 0.001 vs. VASH1 treated DM group. *DM *diabetes mellitus.

We further determined whether the inhibition of Ang1 or VEGF affects the beneficial effects of VASH1. Nerve-induced erectile function studies revealed that inhibition of Ang1 or VEGF with soluble Tie2-Fc or VEGF-trap diminished the improvement in the erectile function by VASH1 in the diabetic mice (Fig. 7a–c). Furthermore, immunohistochemical staining of cavernous tissues demonstrated that the restoration of endothelial cell and pericyte content induced by VASH1 protein also significantly declined after treatment with soluble Tie2-Fc or VEGF-trap (Fig. 7d–f).

## Discussion

Here, we studied the role of VASH1 in a mouse model of diabetic ED. Local delivery of VASH1 protein into the corpus cavernosum of diabetic mice rescued erectile function by enhancing eNOS phosphorylation and by restoring endothelial cells, pericytes, and endothelial cell to cell junction proteins. The major mechanism accountable for VASH1-mediated promotion of cavernous angiogenesis and restoration of erectile function in diabetic mice is the induction of angiogenic factors, such as Ang1 and VEGF.

It was reported that the expression of VASH1 is induced in endothelial cells by angiogenic factors at the site of angiogenesis^24^. However, VASH1 is also known to be detectable in endothelial cells in quiescent state^25^. Similar to this finding, we demonstrated the expression of VASH1 in cavernous endothelial cells. Moreover, the cavernous expression of VASH1 was profoundly downregulated in diabetic conditions.

In the present study, diminished cavernous endothelial area was found in the diabetic group than in the control group. Local administration of VASH1 protein into the corpus cavernosum completely reinstated cavernous endothelial cell content in the diabetic group. The previous research reported that knockdown of VASH1 in endothelial cells induces premature senescence through an increase in the production of reactive oxygen species and a decrease in antioxidant enzyme^26^. The senescent endothelial cells are more prone to cell death in pathological conditions in vitro, such as exposure to H_2_O_2_ and serum deprivation^27^. On the contrary, overexpression of VASH1 in endothelial cells showed resistance to premature cellular senescence and cell death^26^, supporting the role of VASH1 in stress tolerance and vascular protection.

The lack of pericyte coverage in microvessels is one of the major mechanisms responsible for diabetic angiopathies, such as diabetic retinopathy^28^ and diabetic ED^5^. Although the effects of VASH1 on mural cells were described in tumor vessels^29^, i.e., the normalization of tumor vasculature by intensifying pericyte coverage, its role in the non-tumor blood vessel is still unraveled. In the present study, VASH1 protein also significantly restored pericyte content in the diabetic mice.

Endothelial cell–cell barrier-genesis is critical for regulating vascular permeability and plays a crucial function during blood vessel formation and maturation^21^. Premature endothelial cell senescence induced by knockdown of VAHS1 shows impairments in the integrity of cell–cell junctions^30^. Knockdown of VASH1 decreased the expression of zonula occludens-1, a tight junction protein, in endothelial cells and increased transmigration of Lewis lung carcinoma cells across the endothelial cell monolayer. Moreover, the overexpression of VASH1 rescued the expression of zonula occludens-1^30^. Comparable to these results, we demonstrated that intracavernous treatment with VASH1 protein significantly reestablished cavernous endothelial cell–cell junction proteins in the diabetic mice. However, it remains to be further elucidated how VASH1 regulates endothelial cell–cell junction integrity.

In this study, VASH1 restored the expression of Ang-1 and VEGF, which were downregulated in the diabetic penis. Similar to our finding, Ang1 expression was significantly decreased in the kidney of diabetic mice compared with the control mice and was further decreased in the diabetic VASH1 heterozygous knockout (VASH1^+/−^) mice^14^.

Although VEGF is a potent angiogenic factor, but by itself initiates leaky and unstable blood vessel formation^31^. In comparison, Ang1, the ligand for tyrosine kinase with immunoglobulin and epidermal growth factor homology domain-2 (Tie2), has a major role in vascular maturation and stabilization^32^. Ang1 also ameliorates VEGF induced inflammation while having an additive influence on angiogenesis^33^. We previously reported in an animal model of hypercholesterolemic ED that adenoviral-mediated combined Ang1 and VEGF gene therapy exerted a synergism on angiogenesis and on recovery of erectile function compared with that of either therapy alone^34^. Moreover, our recent study demonstrated in diabetic mice that synthetic Ang1 protein also restored the integrity of endothelial cell–cell junction^22^. These findings suggest that induction of angiogenic factors is one of the major mechanisms by which VASH1 induces cavernous angiogenesis and restores erectile function in the diabetic mice. Inhibition studies with soluble Tie2-Fc or VEGF-trap further supported the role of VASH1-mediated up-regulation of angiogenic factors on diabetic ED.

## Conclusions

Our findings demonstrate an unanticipated and unique function of VASH1 in the diabetic ED. The intracavernous injection of VASH1 protein enhanced cavernous angiogenesis and restored erectile function by boosting angiogenic factors expression in diabetic mice. As far as we know, this is the first study demonstrating that VASH1 is proangiogenic in the diabetic penis. Further investigations are necessary to discover the efficacy of VASH1 in different disease models for ED.

## Materials and methods

### Study design

The primary aim of this study is to explore the role of VASH1 on cavernous vascular integrity and erectile function. For this purpose, we used VASH1 homozygous-knockout (VASH1^−/−^) mice or VASH1 protein.

### Animals and treatments

Age-matched, pathogen-free, C57BL/6 mice were obtained from Orient Bio (Gyeonggi, South Korea) and two-month-old male mice were used for this study. Animal care and all experimental methods were handled following with the approval and guidelines of the INHA Institutional Animal Care and Use Committee (INHA IACUC) of the Medical School of Inha University (IACUC No. INHA 180727-581). Diabetes was provoked by intraperitoneal injection of repeated low doses of STZ (50 mg/kg body weight in 0.1 M citrate buffer, pH 4.5) for 5 sequential days as described previously^35^. Animals were considered as diabetic if the nonfasting glucose levels are greater than 300 mg/dL.

Eight weeks after diabetes was induced, the mice were anesthetized with intramuscular injections of ketamine (100 mg/kg) and xylazine (5 mg/kg) and placed supine on a thermoregulated surgical table. The penis was exposed by use of a sterile technique^34, 35^. To examine the effectiveness of VASH1 protein, the mice were then distributed into four groups (N = 6 per group): age-matched controls and STZ-induced diabetic mice receiving sequential intracavernous injections of PBS (days − 3 and 0; 20 μL) or VASH1 protein (days -3 and 0; 1 µg or 4 µg in 20 μL of PBS, respectively). A 30-gauge insulin syringe was used to deliver PBS or VASH1 protein into the midportion of the corpus cavernosum. The incision was closed with 6-O Vicryl (polyglactin 910) sutures. Erectile function was evaluated by electrical cavernous nerve stimulation at 2 weeks after treatment. A separate group of animals was used for histologic examination and biochemical study.

For the inhibition study with soluble Tie2 protein (sTie2-Fc) or VEGF trap, the mice were divided into five groups: age-matched controls and STZ-induced diabetic mice receiving repeated intracavernous injections of PBS (days − 3 and 0; 20 μL) + dimeric-Fc, VASH1 protein (days − 3 and 0; 4 µg in 20 μL of PBS), VASH1 protein + sTie2-Fc (4 μg/20 μL, R&D Systems, Minneapolis, MN, USA), or VASH1 protein + VEGF trap (4 mg/kg, R&D Systems). The sTie2-Fc or VEGF trap was administered subcutaneously shortly before intracavernous-injection of VASH1 protein to investigate the role of Ang1 or VEGF in VASH1-mediated cavernous angiogenesis and erectile function.

### Human corpus cavernosum tissue

Human corpus cavernosum tissue samples were obtained from a 21-year-old patient with congenital penile curvature who had normal erectile function during reconstructive penile surgery and a 56-year-old patient with diabetic ED during penile prosthesis implantation. All tissue donors provided informed consent, and the experiments were approved by the internal review board of Inha University.

### Measurement of erectile function

The mice from each group were anesthetized with ketamine (100 mg/kg) and xylazine (5 mg/kg) intramuscularly. Erectile function was measured as described previously^35^. Briefly, the bladder and prostate exposed through a midline abdominal incision. The major pelvic ganglion and cavernous nerve were identified posterolateral to the prostate on one side, and bipolar platinum wire electrodes were placed around the cavernous nerve for electrical stimulation. The penis skin was denuded and a 26-gauge needle filled with 250 U/mL of heparin inserted into one side of the corpus cavernosum for monitoring ICP with a Statham P23 pressure transducer connected to a computerized system for data acquisition (Biopac Systems, Goleta, CA, USA), which was interfaced to a personal computer for recording and data analysis^35^. Stimulation parameters were 5 V at a frequency of 12 Hz, a pulse width of 1 ms, and a duration of 1 min. During tumescence, the maximal ICP was recorded. The total ICP was determined by the area under the curve from the beginning of cavernous nerve stimulation to a point 20 s after stimulus termination. Systemic blood pressure was measured by using a noninvasive tail-cuff system (Visitech systems, Apex, NC, USA). The ratios of maximal ICP and total ICP (area under the curve) to MSBP were calculated to adjust for variations in systemic blood pressure^35^.

### Histological examinations

The penis tissue (N = 6 per group) was fixed in 4% paraformaldehyde for 24 h at 4 °C as described previously^10^. Frozen tissue sections (20-μm thick) were incubated with antibodies to VASH1 (Santa Cruz Biotechnology Inc., Dallas, TX USA; 1:50), FITC-conjugated isolectin B4 (IB4, Sigma-Aldrich, MO, USA; 1:50), PECAM-1 (Millipore, Temecula, CA, USA; 1:50), NG2 (Millipore; 1:50), phospho-eNOS (Ser1177, Cell Signaling, MA, USA; 1:50), occludin (NOVUS Biologicals, Centennial, CO, USA; 1:50), claudin-1 (Thermo Fisher, Waltham, Massachusetts, U.S. 1:50), Ang1 (Abcam, Cambridge, U.K.; 1:50), or VEGF (Santa Cruz Biotechnology; 1:50) at 4 °C overnight. After 4 times washes with PBS, the sections were incubated Rhodamine (TRITC) AffiniPure Goat Anti-Armenian Hamster IgG (H + L) antibody or Fluorescein (FITC) AffiniPure F(ab′)_2_ Fragment Donkey Anti-Rabbit IgG (H + L) antibody (Jackson ImmunoResearch Laboratories, Inc., PA, USA; 1:50) for 90 min at room temperature. Mounting medium containing 4,6-diamidino-2-phenylindole (DAPI; Vector Laboratories Inc., Burlingame, CA, USA) was applied to the samples and nuclei were labeled when appropriate. Signals and digital images visualized and obtained with a confocal microscope (FV1000, Olympus, Tokyo, Japan). Quantitative analysis of histologic examinations was processed with an image analyzer system (National Institutes of Health [NIH] Image J 1.34, http://rsbweb.nih.gov/ij/) in double blinded manner.

### Western blot analysis

Equal amounts of protein (40 µg per lane) were electrophoresed on sodium dodecylsulfate-polyacrylamide gels (8–15%), transferred to polyvinylidene difluoride membrane. After the gel transferred to a blot, razor blade and ruler were used to cut the blot into strips corresponding to the size of the protein of interest then probed with antibodies to phospho-eNOS (Ser1177, Cell Signaling; 1:1000), eNOS (BD biosciences, California, USA; 1:1000), Ang1 (Abcam; 1:1000), VEGF (Santa Cruz Biotechnology; 1:1000), or β-actin (Santa Cruz Biotechnology; 1:6000). The results were quantified by densitometry (N = 4 per group).

### Statistical analysis

The results are expressed as mean ± SE. For parametric data, intergroup comparisons calculated by one-way ANOVA followed by Newman–Keuls post hoc tests. We used the Mann–Whitney *U* test or Kruskal–Wallis test to compare nonparametric data. The result considered significant if probability values less than 5%. SigmaStat 3.11 software (Systat Software) used for statistical analyses.

## Supplementary Information

Supplementary Information.

## Abbreviations

Ang1: Angiopoietin-1
Ang4: Angiopoietin-4
bFGF: Basic fibroblast growth factor
DAPI: 4,6-Diamidino-2-phenylindole
ED: Erectile dysfunction
eNOS: Endothelial nitric oxide synthase
ICP: Intracavernous pressure
MSBP: Mean systolic blood pressure
NG2: Neuron-glial antigen 2
NO: Nitric oxide
PECAM-1: Platelet/endothelial adhesion molecule 1
STZ: Streptozotocin
VASH1: Vasohibin-1
VEGF: Vascular endothelial growth factor
WT: Wild-type

## References

[1] Kendirci M, Nowfar S, Hellstrom WJ (2005). The impact of vascular risk factors on erectile function. Drugs Today (Barc)..

[2] Kouidrat Y (2017). High prevalence of erectile dysfunction in diabetes: A systematic review and meta-analysis of 145 studies. Diabet. Med..

[3] Kamenov ZA (2015). A comprehensive review of erectile dysfunction in men with diabetes. Exp. Clin. Endocrinol. Diabetes..

[4] Andersson K-E (2011). Mechanisms of penile erection and basis for pharmacological treatment of erectile dysfunction. Pharmacol.. Rev..

[5] Yin GN (2015). The pericyte as a cellular regulator of penile erection and a novel therapeutic target for erectile dysfunction. Sci. Rep..

[6] Angulo J (2010). Diabetes exacerbates the functional deficiency of NO/cGMP pathway associated with erectile dysfunction in human corpus cavernosum and penile arteries. J. Sex. Med..

[7] Musicki B, Burnett AL (2007). Endothelial dysfunction in diabetic erectile dysfunction. Int. J. Impot. Res..

[8] Ryu J-K, Suh J-K, Burnett AL (2017). Research in pharmacotherapy for erectile dysfunction. Transl. Androl. Urol..

[9] Dall'Era JE (2008). Vascular endothelial growth factor (VEGF) gene therapy using a nonviral gene delivery system improves erectile function in a diabetic rat model. Int. J. Impot.. Res..

[10] Yin GN (2018). Pericyte-derived Dickkopf2 regenerates damaged penile neurovasculature through an angiopoietin-1-Tie2 pathway. Diabetes.

[11] Kwon MH (2013). Effect of intracavernous administration of angiopoietin-4 on erectile function in the streptozotocin-induced diabetic mouse. J. Sex. Med..

[12] Suetomi T, Hisasue S, Sato Y, Tabata Y, Akaza H, Tsukamoto T (2005). Effect of basic fibroblast growth factor incorporating gelatin microspheres on erectile function in the diabetic rat. J. Urol..

[13] Shen J, Yang X, Xiao WH, Hackett SF, Sato Y, Campochiaro PA (2006). Vasohibin is up-regulated by VEGF in the retina and suppresses VEGF receptor 2 and retinal neovascularization. FASEB J..

[14] Nasu T (2009). Vasohibin-1, a negative feedback regulator of angiogenesis, ameliorates renal alterations in a mouse model of diabetic nephropathy. Diabetes.

[15] Yoshinaga K (2011). Roles of intrinsic angiogenesis inhibitor, vasohibin, in cervical carcinomas. Cancer Sci..

[16] Kitajima T (2014). Vasohibin-1 increases the malignant potential of colorectal cancer and is a biomarker of poor prognosis. Anticancer Res..

[17] Kanomata N, Sato Y, Miyaji Y, Nagai A, Moriya T (2013). Vasohibin1 is a new predictor of disease-free survival in operated patients with renal cell carcinoma. J. Clin. Pathol..

[18] Kosaka T (2013). The prognostic significance of vasohibin-1 expression in patients with prostate cancer. Br. J. Cancer..

[19] Hinamoto N (2014). Exacerbation of diabetic renal alterations in mice lacking vasohibin-1. PLoS ONE.

[20] Tanimura S (2019). Renal tubular injury exacerbated by vasohibin-1 deficiency in a murine cisplatin-induced acute kidney injury model. Am. J. Physiol. Ren. Physiol..

[21] Liebner S, Cavallaro U, Dejana E (2006). The multiple languages of endothelial cell-to-cell communication. Arterioscler. Thromb. Vasc. Biol..

[22] Ryu JK (2013). Erectile dysfunction precedes other systemic vascular diseases due to incompetent cavernous endothelial cell–cell junctions. J. Urol..

[23] Hurt KJ (2002). Akt-dependent phosphorylation of endothelial nitric-oxide synthase mediates penile erection. Proc. Natl. Acad. Sci. U. S. A..

[24] Watanabe K (2004). Vasohibin as an endothelium-derived negative feedback regulator of angiogenesis. J. Clin. Investig..

[25] Abe M, Sato Y (2001). cDNA microarray analysis of the gene expression profile of VEGF-induced human umbilical vein endothelial cells. Angiogenesis.

[26] Miyashita H (2012). Angiogenesis inhibitor vasohibin-1 enhances stress resistance of endothelial cells via induction of SOD2 and SIRT1. PLoS ONE.

[27] Alon T, Hemo I, Itin A, Pe’er J, Stone J, Keshet E (1995). Vascular endothelial growth factor acts as a survival factor for newly formed retinal vessels and has implications for retinopathy of prematurity. Nat. Med..

[28] Fu D (2012). Mechanisms of modified LDL-induced pericyte loss and retinal injury in diabetic retinopathy. Diabetologia.

[29] Horie S (2016). Distinctive role of vasohibin-1A and its splicing variant vasohibin-1B in tumor angiogenesis. Cancer Gene Ther..

[30] Ito S, Miyashita H, Suzuki Y, Kobayashi M, Satomi S, Sato Y (2013). Enhanced cancer metastasis in mice deficient in vasohibin-1 gene. PLoS ONE.

[31] Lee RJ, Springer ML, Blanco-Bose WE, Shaw R, Ursell PC, Blau HM (2000). VEGF gene delivery to myocardium: Deleterious effects of unregulated expression. Circulation.

[32] Thurston G (1999). Leakage-resistant blood vessels in mice transgenically overexpressing angiopoietin-1. Science.

[33] Chae JK (2000). Coadministration of angiopoietin-1 and vascular endothelial growth factor enhances collateral vascularization. Arterioscler. Thromb. Vasc. Biol..

[34] Ryu JK (2006). Combined angiopoietin-1 and vascular endothelial growth factor gene transfer restores cavernous angiogenesis and erectile function in a rat model of hypercholesterolemia. Mol. Ther..

[35] Jin HR (2009). Functional and morphologic characterizations of the diabetic mouse corpus cavernosum: Comparison of a multiple low-dose and a single high-dose streptozotocin protocols. J. Sex. Med..

