# Supplementary material for: Vasohibin-1 rescues erectile function through up-regulation of angiogenic factors in the diabetic mice
Source: Sci Rep. 2021 Jan 13;11:1114. doi: 10.1038/s41598-020-80925-7 (PMC7807034; doi:10.1038/s41598-020-80925-7)

**Vasohibin-1 rescues erectile function through up-regulation of angiogenic factors in the diabetic mice**

Kang-Moon Song^1, †^, Woo Jean Kim^1,2, †^, Min-Ji Choi^1^, Ki-Dong Kwon^1^, Anita Limanjaya^1^, Kalyan Ghatak^1^, Jiyeon Ock^1^,

Guo Nan Yin^1^, Yasufumi Sato^3^, Soon-Sun Hong^4^, Ji-Kan Ryu^1, *^, and Jun-Kyu Suh^1, *^

^1^National Research Center for Sexual Medicine and Department of Urology, Inha University School of Medicine, Incheon 22332, Republic of Korea

^2^Department of Anatomy, Kosin University College of Medicine, Busan 49267, Republic of Korea

^3^Department of Vascular Biology, Institute of Development, Aging and Cancer, Tohoku University, Sendai 980-8575, Japan

^4^Department of Drug Development, Inha University School of Medicine, Incheon 22332, Republic of Korea

^†^Kang-Moon Song and Woo-Jean Kim contributed equally to this study.

^*^Correspondence to Ji-Kan Ryu, MD, PhD and Jun-Kyu Suh, MD, PhD

Ji-Kan Ryu, MD, PhD

National Research Center for Sexual Medicine and Department of Urology

Inha University School of Medicine

27, Inhang-Ro, Jung-Gu, Incheon 22332

Republic of Korea

Tel: 82-32-890-3505; Fax: 82-32-890-3099

E-mail: rjk0929@inha.ac.kr

Jun-Kyu Suh, MD, PhD

National Research Center for Sexual Medicine and Department of Urology

Inha University School of Medicine

27, Inhang-Ro, Jung-Gu, Incheon 22332

Republic of Korea

Tel: 82-32-890-3441, Fax: 82-32-890-3097

E-mail: [jksuh@inha.ac.kr](mailto:jksuh@inha.ac.kr)

| **Supplemental Table 1** Physiologic and metabolic parameters for Figure 2 | | | |  |  |
| --- | --- | --- | --- | --- | --- |
|  | Wild type | VASH1 KO |  |  |  |
| Body weight (g) | 25.8 ± 0.4 | 24.6 ± 0.7 |  |  |  |
| Postprandial glucose (mg/dL) | 121.2 ± 8.3 | 118.7 ± 9.5 |  |  |  |
| Fasting glucose (mg/dL) | 98.8 ± 7.7 | 103.4 ± 8.8 |  |  |  |
| MSBP (cm H2O) | 148.9 ± 3.1 | 140.8 ± 5.8 |  |  |  |
| Values are the mean ± standard error from N = 6 animals per group. MSBP = mean systolic blood pressure; STZ = streptozotocin;  VASH1-KO = vasohibin-1 knockout. | | |  |  |  |
|  |  |  |  |  |  |
|  |  |  |  |  |  |
| **Supplemental Table 2** Physiologic and metabolic parameters for Figure 3 | | | | |  |
|  |  | STZ-induced diabetic mice | | |  |
|  | Control | PBS | VASH1 1μL | VASH1 4μL |  |
| Body weight (g) | 26.7 ± 0.3 | 22.8 ± 0.2** | 21.9 ± 0.3** | 22.3 ± 0.2** |  |
| Postprandial glucose (mg/dL) | 131.8 ± 5.7 | 550.0 ± 9.7*** | 559.7 ± 12.8*** | 555.8 ± 10.4*** |  |
| Fasting glucose (mg/dL) | 106.8 ± 9.1 | 415.0 ± 28.5*** | 388.0 ± 22.5*** | 410.1 ± 19.7*** |  |
| MSBP (cm H2O) | 148.7 ± 0.6 | 141.4 ± 2.9 | 142.4 ± 5.5 | 151.8 ± 2.5 |  |
| Values are the mean ± standard error from N = 6 animals per group. MSBP = mean systolic blood pressure;  STZ = streptozotocin; VASH1 = vasohibin-1. ***P* < 0.01 and ****P*< 0.001 vs. control group. | | | | |  |
|  |  |  |  |  |  |
|  |  |  |  |  |  |
| **Supplemental Table 3** Physiologic and metabolic parameters for Figure 7 | | | | | |
|  |  | STZ-induced diabetic mice | | | |
|  | Control | PBS+Fc | VASH1 | VASH1+sTie2 | VASH1+VEGF trap |
| Body weight (g) | 26.1 ± 0.8 | 21.8 ± 0.4** | 22.6 ± 0.6** | 22.1 ± 0.3** | 22.7 ± 0.9** |
| Postprandial glucose (mg/dL) | 128.8 ± 8.1 | 568.4 ± 8.8*** | 559.7 ± 8.5*** | 570.8 ± 7.1*** | 558.0 ± 9.8*** |
| Fasting glucose (mg/dL) | 97.8 ± 4.6 | 399.0 ± 19.1*** | 404.5 ± 15.2*** | 410.1 ± 14.1*** | 407.8 ± 14.3*** |
| MSBP (cm H2O) | 136.0 ± 1.31 | 141.4 ± 5.2 | 143.2 ± 1.7 | 138.4 ± 3.8 | 140.0 ± 3.2 |
| Values are the mean ± standard error from N = 6 animals per group. MSBP = mean systolic blood pressure; STZ = streptozotocin; VASH1 = vasohibin-1; VEGF = vascular endothelial growth factor. ***P* < 0.01 and ****P*< 0.001 vs. control group. | | | | | |
|  |  |  |  |  |  |


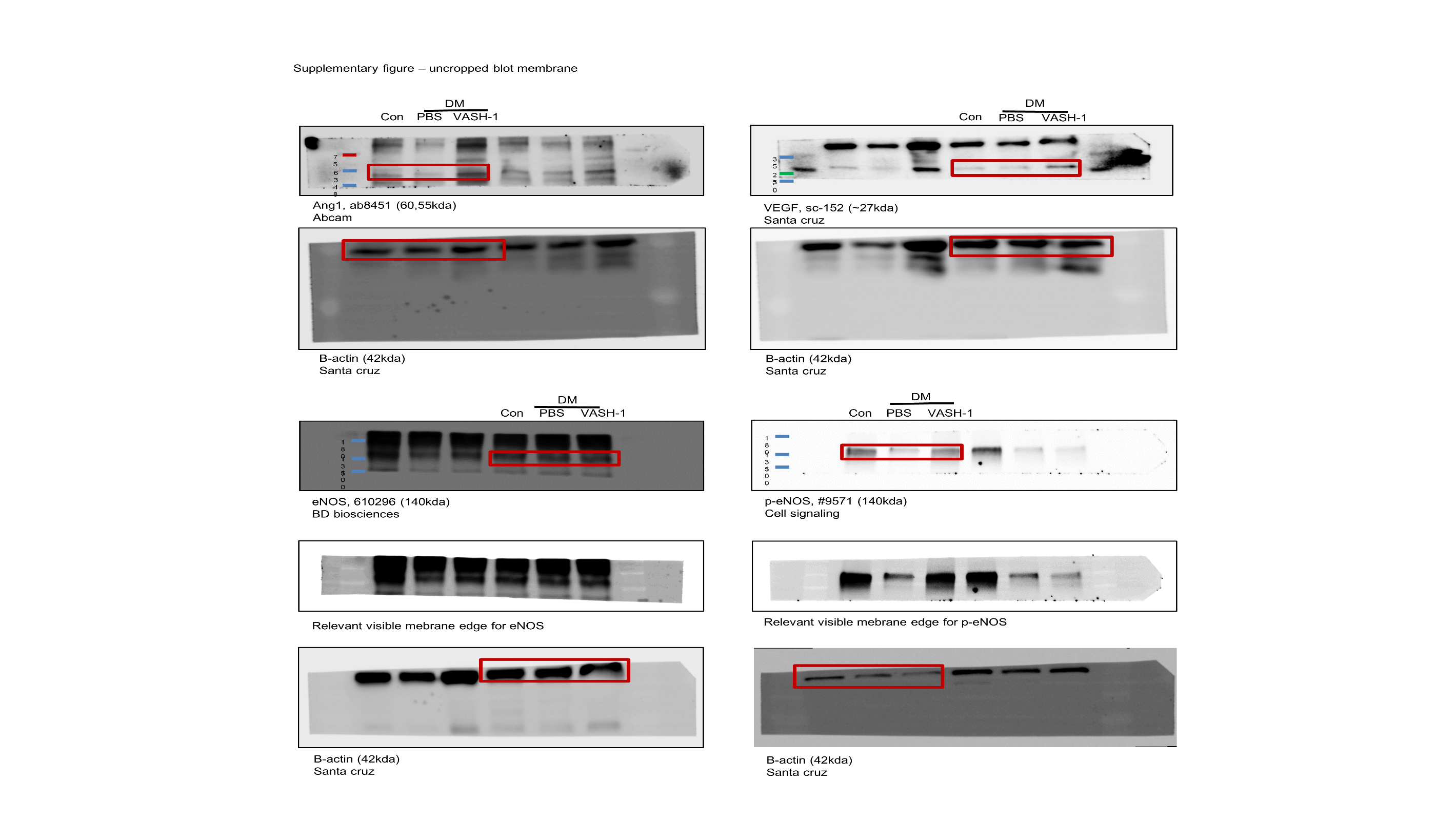

Supplement: Supplementary file 1 — Supplementary Information. [file 41598_2020_80925_MOESM1_ESM.docx]
